# Whole-Organ Magnetic Resonance Imaging Score (WORMS) of the Knee in Professional Soccer Players

**DOI:** 10.1177/19476035251329571

**Published:** 2025-04-05

**Authors:** Goetz Hannes Welsch, Marc Regier, Karl-Heinz Frosch, Milena L. Pachowsky, Frank Oliver Henes, Gerhard Adam, Kai-Jonathan Maas, Malte Lennart Warncke

**Affiliations:** 1 UKE Athleticum—Center for Athletic Medicine, University Medical Center Hamburg-Eppendorf, Hamburg, Germany; 2 Department of Trauma and Orthopedic Surgery, University Medical Center Hamburg-Eppendorf, Hamburg, Germany; 3 Radiologie München, Munich, Germany; 4 BG Hospital Hamburg, Hamburg, Germany; 5 Department of Internal Medicine 3–Rheumatology and Immunology, University Hospital of Erlangen, Friedrich-Alexander-Universität Erlangen-Nürnberg, Erlangen, Germany; 6 Department of Diagnostic and Interventional Radiology and Nuclear Medicine, University Medical Center Hamburg-Eppendorf, Hamburg, Germany

**Keywords:** osteoarthritis, diagnosis, knee joint, whole-organ magnetic resonance imaging score, professional soccer, magnetic resonance imaging, general

## Abstract

**Objective:**

The goal of our study was to assess the prevalence of osteoarthritis in the knee joint of active male professional soccer players by means of the semi-quantitative Whole-Organ Magnetic Resonance Imaging Score (WORMS).

**Design:**

Magnetic resonance imagings (MRIs) of both knees were performed on 85 male professional soccer players during their “medicals” (age = 24 ± 4 years). All baseline data (age, playing position) were obtained. Based on the WORMS, the status of the cartilage and bone in the medial and lateral femoro-tibial joint (MFTJ and LFTJ), as well as the patellofemoral joint was assessed. Menisci and ligaments were evaluated separately. The final score was the sum of all regional scores.

**Results:**

The mean WORMS of the 170 knee joints was 13.3 ± 13.5 points (range = 0-111, achievable scores: 0-290). Cartilage changes were the most common pathologies, observed in 141 of the 170 knee joints. Structural cartilage lesions (WORMS ≥ 2) were observed in 54% of the studied knees. Pathologies of the medial meniscus were associated with cartilage damage of the corresponding MFTJ (r = 0.424, *P* < 0.0001). The same effect was observed for the lateral meniscus and the corresponding LFTJ (r = 0.553, *P* < 0.0001). However, lateral meniscal lesions could be correlated with more other joint pathologies compared to medial meniscal lesions. Total WORMS correlated significantly with increasing age (r = 0.386, *P* = 0.001).

**Conclusion:**

The provided data show the high incidence of knee joint damage in professional football players. In particular, the lateral meniscus appears to play a critical role.

## Introduction

Soccer is the most popular sport in the world with over 300 million active players and more than 200,000 professional players.^1^ The high physical load on the athletes beginning in their adolescence is seen to lead to overuse problems of the joints of the lower extremity. In addition, injuries, especially of the knee joint, are in many cases followed by acute as well as chronic articular cartilage lesions.^2-5^ The prevalence of cartilage changes and following osteoarthritis (OA) is reported very high in former professional athletes; however, available meta-analyses are varying with a wide range from 2.4% to 75%.^2^

Especially early and smaller articular cartilage lesions of the knee joint are not always producing severe symptoms, and professional soccer players are often asymptomatic.^6^ Nevertheless, over time these degenerative changes can lead to reduced athletic performance as well as an early end of careers.^7,8^ In professional soccer, the health of the knee joints can be a crucial factor for the players as well as for the clubs in a very competitive multi-billion dollar business. Hence, a reliable and structured assessment of the status of the knee joints is very important. Magnetic resonance imaging (MRI) is commonly used as the non-invasive gold standard in depicting cartilage lesions of the knee.^9^ High-resolution morphological MRI of the knee is able to evaluate cartilage pathologies as well as changes of the underlying bone and the menisci.^10,11^ Semi-quantitative “Whole-Organ Scores” are tools to objectively assess the recent status of these intra-articular structures. Cartilage, bone marrow edema, synovitis/effusion, periarticular cysts/bursitis, and ligamental and meniscal integrity can be graded based on a numerical system.^12,13^ The “Whole-Organ MRI Score” (WORMS) of the knee^14^ is the most popular and most commonly used semi-quantitative scoring system.^15^ To evaluate the status of a joint and the degree of OA, the different parameters of joint degradation are assessed based on their location in the joint (patellofemoral and femoro-tibial, medial and lateral). The WORMS adds up into the sum of all joint lesions, giving an exact overview of the area and the quantity of these changes. The score correlates to the degree of OA in the specific knee joint.

There are different studies and systematic reviews available reporting on a high rate of cartilage pathologies and knee OA in former professional soccer players.^16-20^ However, so far, no studies report on a structured MR analysis in a defined group of active professional soccer players.^6,16,17^ Some data on knee MRI are up to date only available during the so-called “National Football League (NFL) Combine” in American football.

The goal of our study was to determine the prevalence and the anatomic location of cartilage lesions and osteoarthritic changes in active male professional soccer players of a Bundesliga team by means of the semi-quantitative WORMS.

## Methods

### Subjects

Between January 2012 and July 2020, in 1 professional soccer club (playing first and second national league), 85 players underwent knee MRI. The MRI studies were performed routinely for all newly signed players as part of the medical suitability examination. Inclusion criteria were that the player was at least 18 years old at the time of examination and that a complete 3.0 Tesla MRI set of both knee joints in high-quality resolution was available. Excluded were players who previously suffered a knee injury or had knee surgery within the last 6 months and where invasive treatment for one of the knee joints was required at the time point of the MRI. Players who received joint injections within the last 6 weeks prior to MRI were excluded. At the time point of MRI, no player was assigned to the disabled list. Current conservative therapies such as physiotherapy were not considered as exclusion criteria.

Before the examination, all baseline data (age, height, weight) were obtained. The playing position was documented, and the players were grouped into: (1) goalkeeper; (2) defender, (3) midfielder, and (4) offensive player and striker. The mean age of all 85 players was 24.0 ± 4 years (age ranged from 18 to 36 years). Six goalkeepers, 25 defensive players, 24 midfielders, and 30 offensive players were examined.

The study was approved by the Clinical Institutional Review Board (Ethic Committee of the Medical Chamber of Hamburg), and the background of the study was explained to all participants; written informed consent was obtained from all subjects.

### Imaging

All MRIs were performed on a 3.0T MRI system (Ingenia, Philips, Best, The Netherlands) with a dedicated 16-channel knee coil (dStream, Philips, Best, The Netherlands) at a maximum knee flexion of 5◦ to 10◦. For image analysis, T1-weighted Fast Spin Echo (T1-FSE) sequences and 2D fat-saturated proton-density–weighted fat-saturated fast spin echo (fs-PD-FSE) were obtained (for detailed MR parameters and sequences, see **Table 1**).

**Table 1.** MRI Sequences at 3.0T With a 16-Channel Knee Coil.

Imaging parameters*	T1-FSE cor	fs-PD-FSE sag	fs-PD-FSE tra	fs-PD-FSE cor
TR (ms)	929	6,549	7,833	5,449
TE (ms)	11	27	27	27
Acquisition matrix	600 × 345	408 × 281	520 × 310	400 × 294
Plane spatial resolution (mm^2^)	0.2 × 0.2	0.25 × 0.25	0.25 × 0.25	0.25 × 0.25
FOV (mm)	120	142	130	120
Slice thickness (mm)	2.0	2.5	2.0	2.5
Imaging time (min:s)	4:50	12:27	5:29	5:59
Total imaging time (min:s)	28:45			

* FSE = fast spin echo, fs = fat-suppressed, PD = proton density, TR = repetition time; TE = echo time, FOV = field of view.

### Image Analysis

Altogether, 170 knee MRIs (both knees of 85 players) have been included. All images were reviewed on a dedicated PACS workstation (PACS IW, GE Healthcare, Chicago, Illinois, USA). Image analysis was performed by 1 expert in musculoskeletal imaging (G.H.W., 20 years of experience) and 2 radiologists with 7 years (M.L.W.) and 8 years (K.J.M.) of experience in musculoskeletal imaging. All raters were blinded to the name and possible previous injuries of the players. For reliability analysis, 40 randomly selected players (80 knees) were independently assessed by all raters.

### Quantification of Magnetic Resonance Imaging Findings

The MRI findings of the knee joint were evaluated based on a slightly modified WORMS.^14^ The modification was performed because for all the included professional soccer players, only mild-to-moderate cartilage changes have been found, and no severe osteoarthritic changes were present. Hence, the variable of the original WORMS, bone attrition, did not appear in any of the included players; it was withdrawn from all further analysis. The scoring of all other existing variable of WORMS, the cartilage morphologic features and signal intensity, the bone marrow edema pattern, marginal osteophytes, synovitis/effusion, periarticular cysts/bursitis, anterior and posterior cruciate ligament integrity, medial and lateral collateral ligament integrity, and medial and lateral meniscal changes was performed as previously described.^14^ For the characterization of the extent of regional involvement of cartilage changes, bone marrow edema, subchondral cysts, and osteophytes, the original divisions of the patella in medial and lateral, and the femoral condyles and tibial plateaus in anterior, central, and posterior regions were analyzed. Other pathologic conditions which are not included in the WORMS (e.g., patella bipartite or tendon abnormalities) were also noted.

Cartilage pathologies were graded in very detailed, ranging from 0 (normal thickness and signal) to 6 (diffuse (>75% of the region) full-thickness loss). A further subdivision of minor (≤1) and structural cartilage (≥2) pathologies was performed. This differentiation was implemented as in the WORMS Grade 1 cartilage lesions are defined as “normal thickness, but increased signal in MR images” and the structural partial thickness defects start at Grade 2.

**Figure 1.**
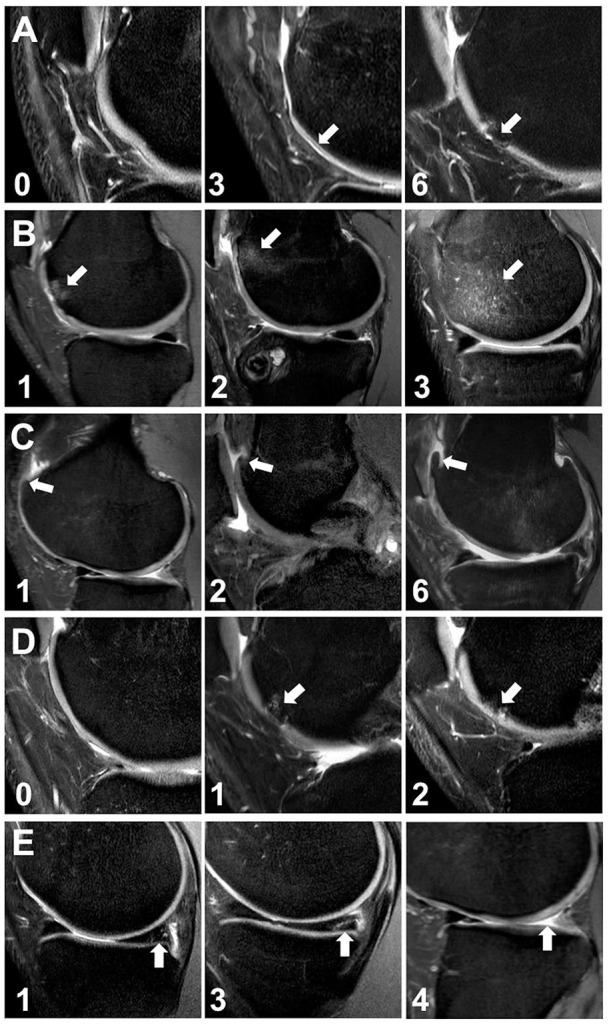
Observed pathologies and corresponding scores. Sagittal PD-weighted FSE of different players (age range = 18-31 years). The regional score for the respective pathology is displayed in the bottom left corner. (A) Cartilage damage at the articular surface of the medial femur (white arrows). (B) Bone marrow edema (white arrows) at the medial femur (C). Osteophytes (white arrows) at the lateral femur. (D) Subchondral cysts (white arrows) at the lateral femur. (E) Degeneration (left) and degenerative tear (middle) of the medial meniscus (white arrows), partial to full resection (posterior aspect) of the lateral meniscus (white arrows) (right).

Bone marrow edema and subchondral cysts were graded from 0 (none) to 3 (present in >50% of the region), whereas osteophytes were depicted in detail based on the WORMS from 0 (none) to 7 (very large). Alterations in meniscal signal intensity were assessed on a 4-level scale on the PDw FSE sequence (0, normal; 1, minor radial or parrot beak tear; 2, non-displaced tear or prior surgical repair; 3, displaced tear or partial resection; 4, complete destruction or complete resection). Different grades of observed pathologies are illustrated in **Figure 1**. The compartments proposed by the original WORMS were combined into larger units: medial and lateral patella and trochlea were summarized as “patellofemoral joint” (PFJ); lateral tibial and lateral femur condyle as “lateral femoro-tibial joint” (LFTJ); and medial tibial and medial femur condyle as “medial femoro-tibial joint” (MFTJ). Finally, all compartments were added together as “whole knee.” Furthermore, we separated the evaluation as well as the results for the right and left knees and in both knees together. To reduce the complexity of the evaluation and to simplify the results for the reader, we mainly provide the results for both knees together. In the evaluation of the right and the left knee joints, we found no significant correlation between the dominant leg and the prevalence of structural abnormalities.

#### Statistical analysis

Statistical analysis was performed by SPSS software version 23 (SPSS Institute, Chicago, Illinois) for Mac (Apple, Cupertino, California). Based on the sample of 170 knee joints (85 players), descriptive statistics employed mean values and standard deviations (SD), and the range of the WORMS score, as well as its different variables. Quantitative evaluation was accomplished by analyses of variance using a 3-way analysis of variance (ANOVA) with a random factor, considering the fact of different measurements within each player. For the correlation between the (1) the different variables of the WORMS score and (2) the variables of the WORMS score and clinical data (age, playing position), a bivariate correlation using the Pearson coefficient was achieved. Regarding intra- and inter-rater reliability, intraclass correlation coefficients (ICCs) were determined based on the independent evaluation of 20 (randomly chosen) cases by 3 readers and all cases by 2 readers (inter-rater assessment) and 20 cases by 2 readers 3 times (intra-rater assessment). The ICC estimates and their 95% confidence intervals (CIs) were calculated based on the mean rating (k = 2 and k = 3) and absolute agreement in a 2-way mixed-effects model. Differences with a *P*-value less than 0.05 were considered statistically significant.

## Results

**Figure 2.**
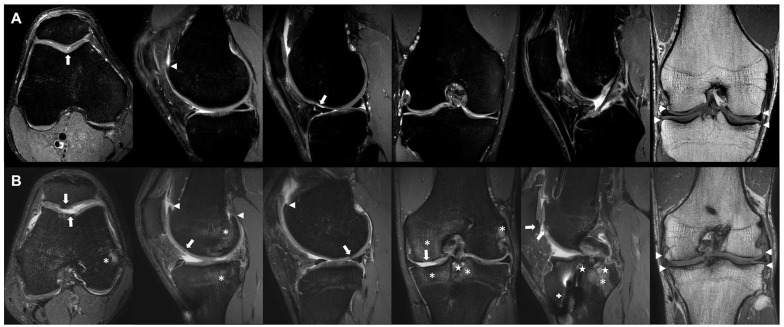
Comparison of 2 professional soccer players’ knee joints. Individual findings are indicated as follows: cartilage damage (white arrows), osteophytes (white arrowheads), bone marrow edema (asterisks), and cysts (stars). (A) Right knee of a 36-year-old professional soccer player with a WORMS of 30. (B) Left knee of a 29-year-old professional soccer player with a WORMS of 111. Note the ACL graft (plus sign).

The mean WORMS of all subjects was 13.3 ± 13.5 points (range = 0-111), where the available range of the assessed WORMS is 0 (no damage) to 290 (most severe damage) points (**Fig. 1**). A direct comparison of 2 different knee joints is shown in **Figure 2**. When looking at the prevalence of the different subscales of the WORMS, cartilage alterations were the most common pathology and could be depicted in 92% of the 85 subjects. Seven players showed no cartilage changes in both knees. Altogether, 29 players (34%) showed either no cartilage or only minor changes (≤ grade 1) in both knees. When looking at the single 170 included knee joints, 29 joints (17%) showed no cartilage alteration and 49 knee joints (29%) showed minor (≤1) cartilage changes, resulting in 78 joints (46%) with no or only minor cartilage pathologies. Vice versa, in 92 knee joints (54%), structural (≥2) cartilage defects could be observed. Besides cartilage pathologies, the depicted joint alteration was bone marrow edema in 59%, osteophytes in 68%, and signs of synovitis in 43% of the included knee joints. As shown in **Table 2**, meniscal lesions could be found in 26% and ligamentous lesions in 8% of the knee joints.

**Table 2.** Prevalence of Knee Abnormalities.

	n = 170 knee joints
Cartilage	Major (WORMS ≥2): 92 (54%) Minor (WORMS =1): 49 (29%) No lesion (WORMS =0): 29 (17%)
Bone marrow edema	100 (59%)
Subchondral cysts	31 (18%)
Osteophytes	115 (68%)
Ligamentous lesions	13 (8%)
Meniscal lesions	Isolated lateral meniscus lesions: 22 (13%) Isolated medial meniscus lesions: 18 (11%) Lesions in both menisci: 4 (2%)
Synovitis	73 (43%)

Cartilage lesions were further subdivided into minor and major lesions. This differentiation was performed as in the WORMS grade 1 cartilage lesions are defined as “normal thickness, but increased signal in MR images” and the structural partial thickness defects start at grade 2. All other features were considered present for a WORMS ≥1. Absolute numbers of knee joints are presented (percentage).

When looking in more detail on the WORMS, from the complete score of 13.3 ± 13.5 points, the cartilage lesions counted for the highest part with a mean of 5.5 ± 5.5 points (*P* < 0.05), followed by osteophytes with 3.8 ± 5.4 points and bone marrow edema with 2.3 ± 3.5 points (**Table 3**).

**Table 3.** Mean WORMS for Individual Features in All Knee Joints and Subjoints.

	*n* = 170 knee joints			
	PFJ	LFTJ	MFTJ	Whole knee
Cartilage	2.0 (±2.8)	2.1 (±2.4)	1.5 (±1.8)	5.5 (±5.5)
Bone marrow edema	1.0 (±1.3)	0.5 (±0.9)	0.4 (±0.8)	2.3 (±2.5)
Subchondral cysts	0.2 (±0.7)	0.1 (±0.2)	0.1 (±0.2)	0.4 (±1.0)
Osteophytes	1.0 (±2.0)	1.4 (±1.8)	1.3 (±1.9)	3.8 (±5.4)
Meniscal lesions		0.3 (±0.7)	0.2 (±0.6)	0.3 (±0.7)
Ligamentous lesions				0.1 (±0.4)
Synovitis				0.7 (±0.8)

The highest mean score for cartilage lesions was observed in the LFTJ. No significant differences were observed for the subregions for all knee joints (*P* > 0.05). Bone marrow edema significantly showed the highest values in the PFJ with 1.0 ± 1.3 points, in comparison to the LFTJ with 0.5 ± 0.9 points and the MFTJ with 0.4 ± 0.8 points (*P* = 0.012). Osteophytes were predominantly present in the MFTJ and LFTJ compared to the PFJ with no statistical significant differences (*P* > 0.05) between these compartments. Mean scores for meniscal lesions did not differ significantly (*P* > 0.05).

**Figure 3.**
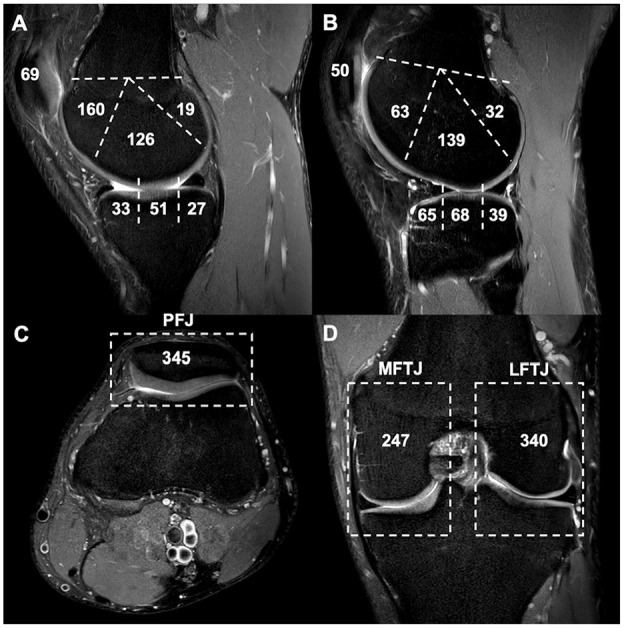
Visual presentation of observed cartilage scores. (A+B) Absolute sum of cartilage scores per subdivision. (C+D) based on (A+B). Overall cartilage scores of the patellofemoral joint (PFJ), the medial femoro-tibial joint (MTFJ), and the lateral femoro-tibial joint (LTFJ).

For further evaluation, we differentiated specific subregions: PFJ, LFTJ, and MFTJ. The highest mean score for cartilage lesions was observed in the LFTJ with 2.1 ± 2.4 points, followed by the PFJ with 2.0 ± 2.8 points and the MFTJ with 1.5 ± 1.8 points **(Table 3, Fig. 3).** The differences between the subregions were not statistically significant (*P* > 0.05) for all knee joints. When looking at the right and the left knees separately, there was a significant difference of mean cartilage values detectable in the comparison between the LFTJ and the MFTJ in the right knee (*P* = 0.041).

**Figure 4.**
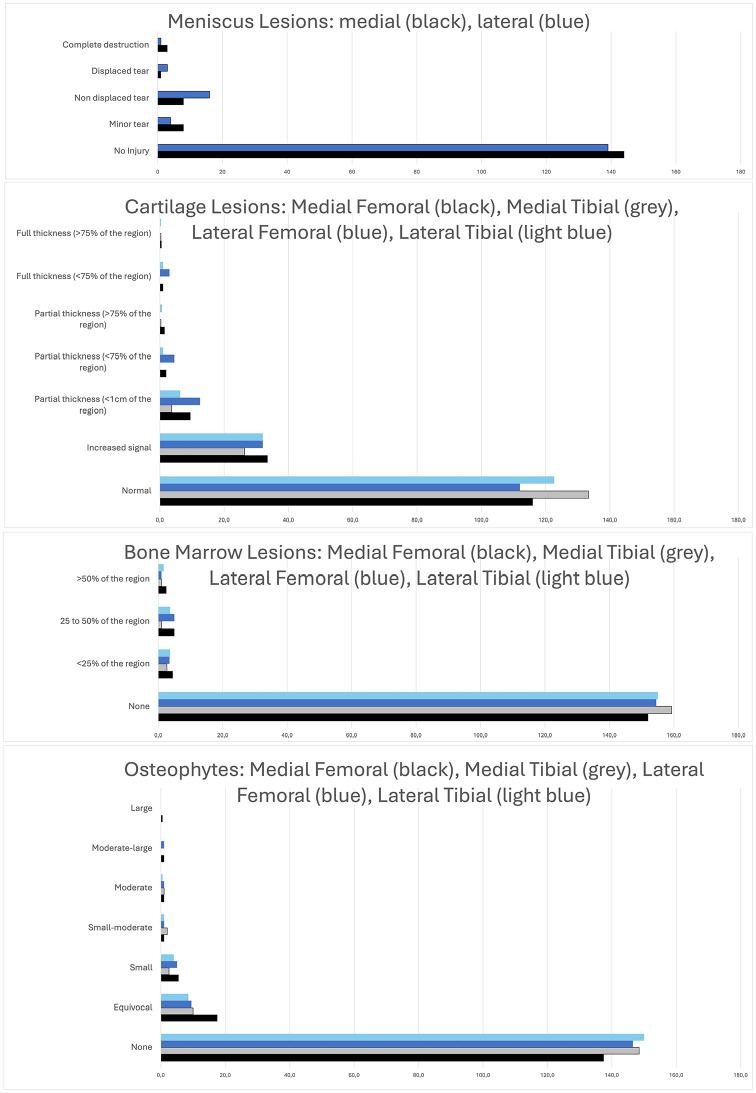
Bar graphs depicting the severity and the frequency of meniscus lesions, cartilage lesions, bone marrow lesions, and osteophytes in the femoro-tibial joint for the medial (black and gray) and the lateral (dark and light blue) aspects of the knee joint.

The osteophytes counted for 1.3 ± 1.9 points in the MFTJ, for 1.4 ± 1.8 points in the LFTJ, and for 1.0 ± 2.0 points in the PFJ with no statistical significant differences (*P* > 0.05). Interestingly, bone marrow edema showed clearly significantly the highest values in the PFJ with 1.0 ± 1.3 points, in comparison to the LFTJ with 0.5 ± 0.9 points and the MFTJ with 0.4 ± 0.8 points (*P* = 0.012). Mean scores for meniscal lesions did not differ significantly between the lateral (0.3 ± 0.7 points) and the medial (0.2 ± 0.6 points) meniscus (*P* = 0.360). We visualized the results for the meniscus, cartilage, bone marrow edema, and osteophytes in relation to the MFTJ and LFTJ in **Figure 4**.

When looking in more detail at the menisci, there was a clear correlation between the severity of the medial meniscal lesions and cartilage lesions of the MFTJ (r = 0.424, *P* < 0.0001) and between lateral meniscal lesions and the LFTJ (r = 0.553, *P* < 0.0001), as well as the PFJ (r = 0.372, *P* < 0.0001) (**Table 4**). Comparably, there were significant correlations between medial meniscal lesions and bone marrow lesions of the MFTJ (r = 0.301, *P* = 0.005) and between lateral meniscal lesions and the LFTJ (r = 0.456, *P* < 0.0001). Regarding the presence of osteophytes, there was only a slight correlation between medial meniscal lesions and osteophytes in the MFTJ (r = 0.227, *P* = 0.037); however, there are clearer correlations between lateral meniscal lesions and osteophytes in all areas of the knee (MFTJ: r = 0.354, *P* = 0.001; LFTJ: r = 0.404, *P* < 0.0001; PFJ: r = 0.283, *P* = 0.009). For the correlation of meniscal lesions and the total WORMS, there was a clearly higher correlation of lateral meniscal lesions to the WORMS (r = 0.468, *P* < 0.0001) compared to the lesions of the medial meniscus to the WORMS (r = 0.284, *P* = 0.008) (**Table 4**).

**Table 4.** Correlations of Medial and Lateral Meniscal Lesions With Cartilage, Bone Marrow Lesions, Osteophytes, Synovitis, and Total WORMS.

		Medial meniscal lesions	Lateral meniscal lesions
Cartilage	MFTJ	**0.424**^a^ <**0.0001**^b^	0.083 0.449
	LFTJ	0.000 1.00	**0.553** <**0.0001**
	PFJ	0.174 0.122	**0.372** <**0.0001**
	Total	**0.224** **0.039**	**0.456** <**0.0001**
Bone marrow lesions	MFTJ	**0.301** **0.005**	**0.234** **0.031**
	LFTJ	0.090 0.412	**0.456** <**0.0001**
	PFJ	0.202 0.064	0.087 0.431
	Total	**0.286** **0.008**	**0.294** **0.006**
Osteophytes	MFTJ	**0.227** **0.003**	**0.354** **0.001**
	LFTJ	0.068 0.536	**0.404** **0.001**
	PFJ	0.211 0.052	**0.283** **0.009**
	Total	0.182 0.096	**0.366** **0.001**
Synovitis		**0.220** **0.043**	**0.258** **0.017**
WORMS total		**0.284** **0.008**	**0.468** <**0.0001**

Significant correlations are printed in bold. No significant correlation was observed between meniscal lesions and subchondral cysts and ligamentous lesions.

a Pearson correlation coefficient.

b Two-sided level of significance.

The players age showed a clear correlation to cartilage pathologies at the PFJ (r = 0.383, *P* < 0.0001), and a slight correlation in the LFTJ (r = 0.221, *P* = 0.042) but not to cartilage pathologies at the MFTJ (*P* > 0.05). Concerning the other evaluated changes in the WORMS, older players showed a higher amount of osteophytes in all subregions (r = 0.360 to 0.392, *P* < 0.0001). The depicted bone marrow edema showed only in the LFTJ a significant correlation to the age of the players (r = 0.259, *P* = 0.017). Regarding the meniscus, only lateral meniscal lesions (r = 0.313, *P* = 0.004) correlated significantly to age. Ligamental lesions, subchondral cysts, and synovitis did not correlate to age. Finally, the total WORMS was significantly higher in older players (r = 0.386, *P* = 0.001). The playing position did not have any significant correlation to the different parameters as assessed in the WORMS (*P* > 0.05).

Inter-rater and intra-rater reliabilities were very high. The ICC (inter-rater reliability) was 0.89 (0.87-0.91) for all 3 raters (*n* = 20) and 0.91 (0.90-0.92) for 2 readers (all cases); the ICC (intra-rater reliability) was comparably high with 0.89 (0.87-0.91).

## Discussion

In this retrospective cross-sectional study, we aimed to investigate knee abnormalities of active professional soccer players by means of a semi-quantitative WORMS.

The most commonly observed knee abnormality was focal chondral defects during the medical examination. In the studied cohort of 85 professional soccer players, 92 knees (54%) reached a WORMS of grade 2 or higher for chondral lesions, indicating a structural cartilage defect. There were no significant differences in the prevalence of cartilage lesions in the respective subregions, whereas bone marrow edema was most frequently found in the PFJ. Concerning the evaluated meniscal lesions, there were clear correlations to cartilage lesions, bone marrow edema, and osteophytes, which were more pronounced for lateral meniscal lesions compared to medial meniscal lesions.

Previous studies report the overall prevalence of focal chondral defects in the knee with 36% and up to 59% among asymptomatic basketball players, volleyball players and long-distance runners.^21-24^ Hirshorn *et al*. evaluated “at-risk” knee joints with MRI during the US NFL Scouting Combine from 2005 till 2007. Out of the total 980 players, 516 underwent MRI (53%). In total, 197 (38.2%) players had full-thickness chondral injuries evident on MRI. A total of 30 players (5.8%) had isolated medial compartment full-thickness chondral injuries, 41 (7.9%) had isolated lateral compartment full-thickness chondral injuries, and 48 (9.3%) had patellofemoral compartment full-thickness chondral damage. A subsequent study by Provencher *et al.* supports these findings.^3^ Out of 2,285 reviewed football players, 63% had chondral lesions of the patella and 34% at the trochlea. These findings are challenged by Brophy *et al*. who evaluated all articular cartilage knee injuries documented in the NFL database from 1992 to 2006. In total, 118 defects were identified. Most lesions were located on the weightbearing surfaces of the medial femoral condyle (54%).^25^ An important contributing factor to this finding could be due to the mean body mass index (BMI) among football players, which is generally higher (31 kg/m^2^) compared to soccer players (23 kg/m^2^).^25,26^

Another study on football players of the NFL Combine reported the lateral compartment at greater risk.^3^ A much higher rate of full-thickness lesions after meniscectomy was observed in the lateral compartment (25%) compared with the medial compartment (6%).^21,27^

The amount of cartilage lesions and also their anatomic distribution varied between the different studies and is roughly comparable to our data. Nevertheless, the inclusion criteria as well as the different cohorts are not comparable. Our study clearly improves previous investigations by including all players, hence providing an unbiased investigation of the prevalence of chondral defects in our cohort. Nevertheless, it has to be pointed out that reporting on the incidence of abnormal structures can lead to false positive results.

Only Stahl *et al*.^22^ and Boeth *et al*.^24^ also used the WORMS to analyze their data. Especially Boeth *et al*.^24^ showed the ability of WORMS to assess knee abnormalities in volleyball players longitudinally, although 2 years seemed to be not a long enough period to depict significant changes over time. In any case, it remains desirable to achieve more longitudinal data in professional sports by semi-quantitative MRI.

Concerning the anatomical distribution of cartilage lesions in correlation to the studied cohorts, the PFJ seems to be one of the most affected areas. In our study, although the differences were not significant, the PFJ and especially the LFTJ showed the highest amount of chondral defects. Whereas cartilage lesions of the PFJ might be induced by the high load during jumping and stop and go, the defects of the LFTJ are seen in clear correlation to meniscal lesions.

The high prevalence of structural chondral defects in asymptomatic soccer players (54% of the studied knees) makes the management of these lesions challenging. Osteophytes were present in 115 knee joints (68%). Almost the same incidence was reported in healthy subjects with a mean age of 62 years.^28^ This finding underlines the high stress on the knee joints of professional soccer players, which leads to early joint degeneration. In our cohort, the severity of osteophytes correlated significantly with age. Osteophytes are a hallmark of and are associated with the progression of OA.^29^ Hence, the semi-quantitative evaluation with WORMS can potentially serve as an indicator of ongoing joint degeneration. Furthermore, follow-up exams could be used to assess the progression rate of OA.

Bone marrow edema was commonly observed in the studied soccer players (59% of the knee joints). The highest prevalence was observed in the PFJ (*P* = 0.012). The signal alterations are not specific but are believed to be caused by intraosseous microtrauma or increased intravascular pressure due to early cartilage defects.^30^ Previous studies reported bone marrow lesions in asymptomatic athletes: Boeth *et al*. found that 61% of asymptomatic adult volleyball players had bone marrow lesions (this study did not specify the prevalence in the subjoints), Major *et al*.^31^ reported a prevalence of 41% in varsity basketball players (in descending frequency: in the medial femoral condyle [7], the patella [3], the lateral femoral condyle [2], and the tibia plateau [2]), and finally Soder *et al*.^32^ found that 50% of 28 asymptomatic 14- to 15-year-old recreational soccer players had bone marrow lesions (in descending frequency: in the medial femoral condyle, the patella, and the tibia plateau). Our results are coherent with the previous published data on the prevalence of bone marrow edema in asymptomatic athletes. Running and repetitive jumping may explain the high prevalence of bone marrow lesions in athletes.^33^

Subchondral cysts were found in 18% of the knee joints. A similar incidence was reported in adult volley ball players.^24^ Guermazi *et al*.^28^ found subchondral cysts in 19% of healthy adults aged between 50 and 60 years. This suggests that soccer players have a higher incidence for subchondral cysts than the general population of the same age. Usually, subchondral cysts are asymptomatic, but they may indicate already degenerative joint disease.^34^ Furthermore, Audrey *et al*. reported that subchondral cysts were present in only 31% of 806 knees with radiographic OA. They concluded that subchondral cysts may be a late pathological feature in OA but should not be considered a cardinal radiographic feature.^34^ This is concordant with our findings. Subchondral cysts showed no association with age.

Meniscal lesions were observed in 26% of the included knee joints. Previous studies have a wide spread of reported incidence of meniscal lesions in adult athletes. Kaplan *et al.* found meniscal lesions in 20% of professional basketball players.^27^ Boeth *et al*.^24^ found meniscal lesions in 61% of 56 adult volleyball players aged 47 ± 5 years. Reinig *et al*.^35^ reported the incidence of 58% for meniscal lesions in college football players. Soder *et al*.^32^ found no meniscal lesions at all in adolescent soccer players. Since meniscal injuries are very common in soccer, our findings appear to be plausible, in comparison with the aforementioned studies.^36^

The meniscus appears to be a crucial anatomical structure for overall joint integrity in athletes. Significant correlations were observed between pathologies of the lateral meniscus and cartilage lesions of the LFTJ, PFJ, bone marrow lesions, osteophytes, synovitis, and total WORMS. By comparison, pathologies of the medial meniscus correlated only significantly with cartilage lesions of the MFTJ, as well as to less areas of bone marrow lesions and osteophytes (**Table 4**). It has been shown that meniscal lesions lead to early-stage OA.^37,38^ The hypothesis that the integrity of the lateral meniscus is very important in soccer players for the prevention of early OA and playing performance is supported by a study by Nawabie *et al*.^39^ in 2017. They reported that return to play time is significantly longer in elite soccer players after lateral meniscectomy compared to medial meniscectomy. In addition, Beaufils and Pujol^40^ stressed the “save the meniscus” approach in the management of traumatic meniscal tear and degenerative lesions in young soccer players to prevent early OA and preserve play time. Gee *et al*.^41^ emphasized in their 2020 published study the importance of careful treatment and management of meniscal lesions in young athletes. Playing position had no significant influence on knee abnormalities.

Our study has several limitations. First, the number of players our cohort is rather small compared to the studies of the NFL Combine; nevertheless, in our study, all players (no matter if they showed knee problems or had knee injuries or surgeries) were included, which increases the meaningfulness of our findings for the prevalence of knee pathologies in professional soccer players. Second, there is no defined gold standard grounding the MR findings, as diagnostic arthroscopy could not have been performed in our study population. Third, we included only players of 1 professional soccer club, which could be another bias. Future studies should include a higher number of players who should be studied in a multi-center approach. Fourth, a longitudinal evaluation at different time points would be desirable to learn more about the natural course of knee abnormalities in professional sports. Fifth, it would be desirable to assess in future studies the functional limitations of soccer players based on their joint pathologies. In addition, the present study shows that the WORMS might need some adaptations for an even better applicability in professional sports, and it has to be pointed out that the high correlation among the different variables is expectable, as these factors are coincidental.

## Conclusion

The provided data show the high incidence of joint damage in professional football players and its distribution in the knee joint. In particular, the lateral meniscus appears to play a critical role and damage correlates highly with lateral compartment joint changes. Our data furthermore suggest that WORMS is applicable in the medical examination of professional soccer players and provides a clinically feasible approach to assess joint health. Future studies have to depict in longitudinal evaluations the individual progression of OA in the knee joints of athletes during their career.
